# Maize/soybean intercropping increases nutrient uptake, crop yield and modifies soil physio-chemical characteristics and enzymatic activities in the subtropical humid region based in Southwest China

**DOI:** 10.1186/s12870-024-05061-0

**Published:** 2024-05-21

**Authors:** Jamal Nasar, Munir Ahmad, Harun Gitari, Li Tang, Yuan Chen, Xun-Bo Zhou

**Affiliations:** 1 Guangxi Key Laboratory of Agro‑Environment and Agro‑Products Safety, Key Laboratory of Crop Cultivation and Physiology, College of Agriculture, Guangxi University, Nanning, 530004 China; 2 College of Plant Protection, Yunnan Agricultural University, Kunming, 650201 China; 3 Department of Agricultural Science and Technology, School of Agriculture and Environmental Sciences, Kenyatta University, P.O. Box 43844-00100, Nairobi, Kenya; 4 College of Resources and Environmental Science, Yunnan Agricultural University, Kunming, 650201 China; 5 Guangxi Academy of Agricultural Sciences, Nanning, 530007 China

**Keywords:** Intercropping, Plant nutrients, Soil nutrients, Soil enzymatic activity, crop yield, Agricultural sustainability

## Abstract

Intercropping, a widely adopted agricultural practice worldwide, aims to increase crop yield, enhance plant nutrient uptake, and optimize the utilization of natural resources, contributing to sustainable farming practices on a global scale. However, the underlying changes in soil physio-chemical characteristics and enzymatic activities, which contribute to crop yield and nutrient uptake in the intercropping systems are largely unknown. Consequently, a two-year (2021–2022) field experiment was conducted on the maize/soybean intercropping practices with/without nitrogen (N) fertilization (i.e., N_0_; 0 N kg ha^−1^ and N_1_; 225 N kg ha^−1^ for maize and 100 N kg ha^−1^ for soybean ) to know whether such cropping system can improve the nutrients uptake and crop yields, soil physio-chemical characteristics, and soil enzymes, which ultimately results in enhanced crop yield. The results revealed that maize intercropping treatments (i.e., N_0_MI and N_1_MI) had higher crop yield, biomass dry matter, and 1000-grain weight of maize than mono-cropping treatments (i.e., N_0_MM, and N_1_MM). Nonetheless, these parameters were optimized in N_1_MI treatments in both years. For instance, N_1_MI produced the maximum grain yield (10,105 and 11,705 kg ha^−1^), biomass dry matter (13,893 and 14,093 kg ha^−1^), and 1000-grain weight (420 and 449 g) of maize in the year 2021 and 2022, respectively. Conversely, soybean intercropping treatments (i.e., N_0_SI and N_1_SI) reduced such yield parameters for soybean. Also, the land equivalent ratio (LER) and land equivalent ratio for N fertilization (LER_N_) values were always greater than 1, showing the intercropping system’s benefits in terms of yield and improved resource usage. Moreover, maize intercropping treatments (i.e., N_0_MI and N_1_MI) and soybean intercropping treatments (i.e., N_0_SI and N_1_SI) significantly (*p* < 0.05) enhanced the nutrient uptake (i.e., N, P, K, Ca, Fe, and Zn) of maize and soybean, however, these nutrients uptakes were more prominent in N_1_MI and N_1_SI treatments of maize and soybean, respectively in both years (2021 and 2022) compared with their mono-cropping treatments. Similarly, maize-soybean intercropping treatments (i.e., N_0_MSI and N_1_MSI) significantly (*p* < 0.05) improved the soil-based N, P, K, NH_4_, NO_3_, and soil organic matter, but, reduced the soil pH. Such maize-soybean intercropping treatments also improved the soil enzymatic activities such as protease (PT), sucrose (SC), acid phosphatase (AP), urease (UE), and catalase (CT) activities. This indicates that maize-soybean intercropping could potentially contribute to higher and better crop yield, enhanced plant nutrient uptake, improved soil nutrient pool, physio-chemical characteristics, and related soil enzymatic activities. Thus, preferring intercropping to mono-cropping could be a preferable choice for ecologically viable agricultural development.

## Introduction

 In China, the demand for food products to satisfy the increasing population is increasing consistently, with agriculture playing a significant role in such scenarios [1]. However, relying heavily on chemical fertilizers for agricultural production is resulting in serious harm to the agricultural ecosystem and environment, with consequences being observed in not only the quantity and quality of agricultural products but also in basic soil fertility [2, 3]. Equally, the long-term intensive sole-cropping leads to resource waste (e.g., nutrients, solar radiation, water, and land) and a marked reduction in agricultural biodiversity [4]. Moreover, the crop’s yield and quality have also been negatively impacted by the low soil fertility caused by mainly terrestrial soil degradation and erosion [5]. Besides, the quantity and quality of fodder produced in grasslands have declined due to excessive grazing and significant soil erosion [6]. Additionally, rapid urbanization significantly reduces the agricultural cultivable land, which threatens the food security of the country [7]. Consequently, the agricultural systems need to be modified to address such challenges. Thus, to improve crop yield, develop a sustainable agricultural production system, achieve better utilization of natural resources (i.e., land and nutrients, etc.), and reduce the risk of high chemical fertilization, establishing a cereal–legume intercropping system could be a better option.

Intercropping, which refers to the co-cultivation of different crops on the same farm-land at the same or different times [8, 9], is an old cropping practice that dates back to ancient civilization and is still widespread [10]. Over the last few years this system has drawn a lot of research interest, because of some of its valuable effects including higher crop yields, efficient exploitation of resources (i.e., nutrients, solar radiation, land and water, agricultural sustainability, environmental safety, and less fertilization requirements [11]. Intercropping, as opposed to mono-cropping, vividly boosts crop yield, plant nutrient uptake, and soil fertility by making efficient use of the natural resources, underlying facilitative root interactions, N_2_ fixation ability of legumes, sharing of nutrients, rhizospheric alteration such as changes in soil physio-chemical properties and enzymatic activity, root exudation, rich abundance of beneficial microbes, and some unknown mechanisms [12–14].

It has been documented that intercropping two different crops drastically improved the crop yield and the uptake of nutrients particularly N, P & K mostly due to the underlying soil modification fostered by intercrop roots [15–17]. For example, the underlying rhizospheric alterations, such as better soil nutrient availability, changes in the soil physio-chemical properties, enzymatic activities, and microbial communities, were mostly responsible for the improved yield and nutrient uptake in maize soybean intercropping [18, 19]. Maize-alfalfa intercropping has also been demonstrated to increase the amount of nitrogen in the soil and increase the uptake of nutrients by the maize crops through facilitative root interaction since alfalfa is a legume crop and can fix atmospheric nitrogen (N_2_), this ultimately increases crop yield [20]. Equally, the rhizospheric modifications and root-releasing substances (such as phosphatases, phytase, and carboxylates) in intercropping help mineralize and mobilize the P in the soil to plant available form. Such a move increases the plant’s total P concentration and its uptake and subsequently increases crop yield [21, 22]. In comparison to their mono-cropping systems, cereal-legume intercropping have shown increases in crop yields, plant nutrient uptake (N, P, and K), and soil nutrient status [18, 23, 24]. Such enhancement of plant nutrient uptake and soil fertility in the intercropping (i.e., cereal-legume intercropping) is closely linked to root-releasing chemicals and below-ground interspecific root interactions, which likely boost plant nutrient uptake as well as the nutritional quality and yield of the crops [25, 26].

The Guangxi Province in Southern China is a typical subtropical monsoon humid region with abundant rainfall, whose distribution is uneven both temporally and spatially. Maize is a major crop in this region, grown twice a year (i.e., in spring and autumn) called a double cropping system, and accounts for 18.4–20.4% of the total cultivated area [27]. Maize is sometimes referred to as the Queen of Cereals because of its high crop yield and forage production ability [28]. Conversely, soybean (*Glycine max* L), a perennial grain legume, is valued for having a high protein, vitamin, and mineral content [29]. It also helps in improving soil fertility and health by replenishing it with nutrients, because of its restorative and nitrogen fixation ability [30]. Although maize crop requires high N for the optimum yield, increased use of N fertilization for maize production in such intensive double cropping systems results in no significant gainful effect on grain yield, nutrient uptake, and agricultural soil improvement but rather it results in high costs and wasted resources [27].

Previously, studies on improving crop production, and soil fertility with less chemical inputs including optimization of N fertilization rate for different crop species [31], conventional breeding and genetic modification [32], or agronomy practices [16], while the importance of intercropping is often neglected in this area. Nevertheless, the use of chemical fertilizers for agricultural production affects the agricultural land and its fertility in a multifaceted way, threatens both the quality of crop yield and the environment, and also adversely effects the sustainable development of agricultural land [29, 33]. Also, the high-yielding crop varieties often have lower nutrient contents, which lowers crop nutritional value [34]. Consequently, integrating maize into intercropping systems involving soybeans would not only provide an environmentally pleasant, sustainable, and auspicious agricultural system for future advances, but it also increases the nutrient value of the crops and ensures the regional food demand and nutritional quality of the fodder industry. It is noteworthy that maize-soybean intercropping has been extensively carried out to improve soil health, crop and forage productivity, and the utilization of natural resources and nutrients, especially N, P, and K [14, 16, 18]. However, there is a dearth of information regarding how intercropping influences the other plant nutrients, including calcium (Ca), zinc (Zn), magnesium (Mg), iron (Fe), and manganese (Mn), which are some of the nutrients required for the plant growth and development and often lacking in human nutrition [35]. Even though Fe and Zn continue to be the most researched micronutrients, Ca, Mg, and Mn have received relatively little attention [16]. Nonetheless, all these plant nutrients are normally studied for crops grown under monoculture. However, both the aforementioned macro and micro plant nutrients in maize-soybean intercropping particularly in the subtropical humid region of Guangxi Province are rarely understood. Hence, it is crucial to ascertain whether planting maize and soybeans together in intercropping may improve or balance soil and plant nutrient fertility while simultaneously producing superior crop yields.

Therefore, the purpose of this study was to examine the effects of intercropping maize and soybeans on crop yield, plant nutrient contents, soil physio-chemical properties, and enzymatic activities under various nitrogen fertilizers. It also aimed to elucidate the underlying mechanisms that affect changes in plant nutrient content and production. We hypothesized that by modifying the physio-chemical properties and enzymatic activities of the soil, maize-soybean intercropping might enhance the nutrient uptake of plants and increase crop yield. The central objective of this research was to examine whether intercropping, with or without N fertilizer application, would enhance plant production and nutrient uptake by changing soil physio-chemical properties and enzymatic activity.

## Materials and methods

The field experiment was conducted for two consecutive years in 2021–2022 at Guangxi University, Guangxi province, China. This area falls in under subtropical monsoon climatic condition having an average rainfall of 1080 mm annually. The soil on the experimental site was loamy in texture with a nearly neutral pH (6.45) and organic matter content of 14.7 g kg^−1^. Additionally, the available N, P, and K in this soil were 64.9, 72.6, and 77.0 mg kg^−1^, respectively.

Maize (Zhengdan 958) and soybean (Gui Chun 15) were planted as maize mono-cropping (MM), soybean mono-cropping (SM), and maize-soybean intercropping (MSI) and treated with two nitrogen levels: N_0_ (0 kg N ha^−1^) and N_1_ (225 kg N ha^−1^ for maize and 100 kg N ha^−1^ for soybean), which was 25% less than the local convention N fertilization. This experiment was designed in a split-plot arrangement with two factors: nitrogen fertilizers levels (NL), which was applied in the main plots and planting patterns (PP) in subplots. The treatments comprised of different treatments: N_0_MM (maize mono-cropping without N fertilization), N_0_MI (maize intercropping without N fertilization), N_1_MM (maize mono-cropping with N fertilization), N_1_MI (maize intercropping with N fertilization), N_0_SM (soybean mono-cropping without N fertilization), N_0_SI (soybean intercropping without N fertilization), N_1_SM (soybean mono-cropping with N fertilization), N_1_SI (soybean intercropping with N fertilization), N_0_MSI (maize/soybean intercropping without N fertilization), N_1_MSI (maize/soybean intercropping with N fertilization). All treatments were repeated four times.

**Fig. 1:**
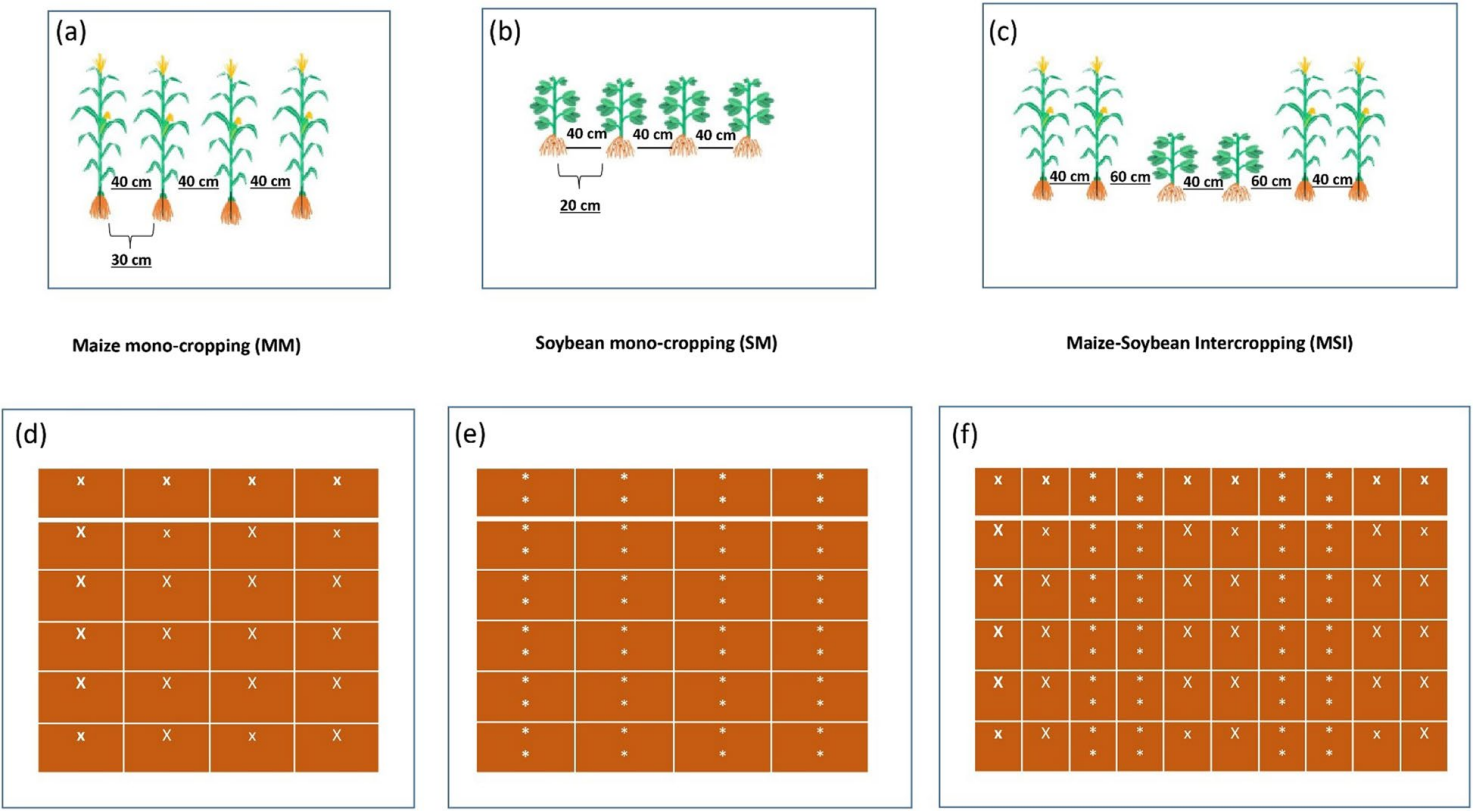
Schematic diagram of the experiment, **a** maize mono-cropping, **b** soybean mono-cropping, **c** maize-soybean intercropping, **d** maize mono-cropping plot representation, **e** soybean mono-cropping plot representation, **f** maize-soybean intercropping representation, 30 and 40 cm represent plant-plant and row-row distance respectively for maize crop, 20 and 40 cm represent plant-plant and row-row distance respectively for soybean crop, 40 and 60 cm represent distance between rows and strips respectively for maize-soybean intercropping, x; maize crop and *; soybean crop

**Fig. 2:**
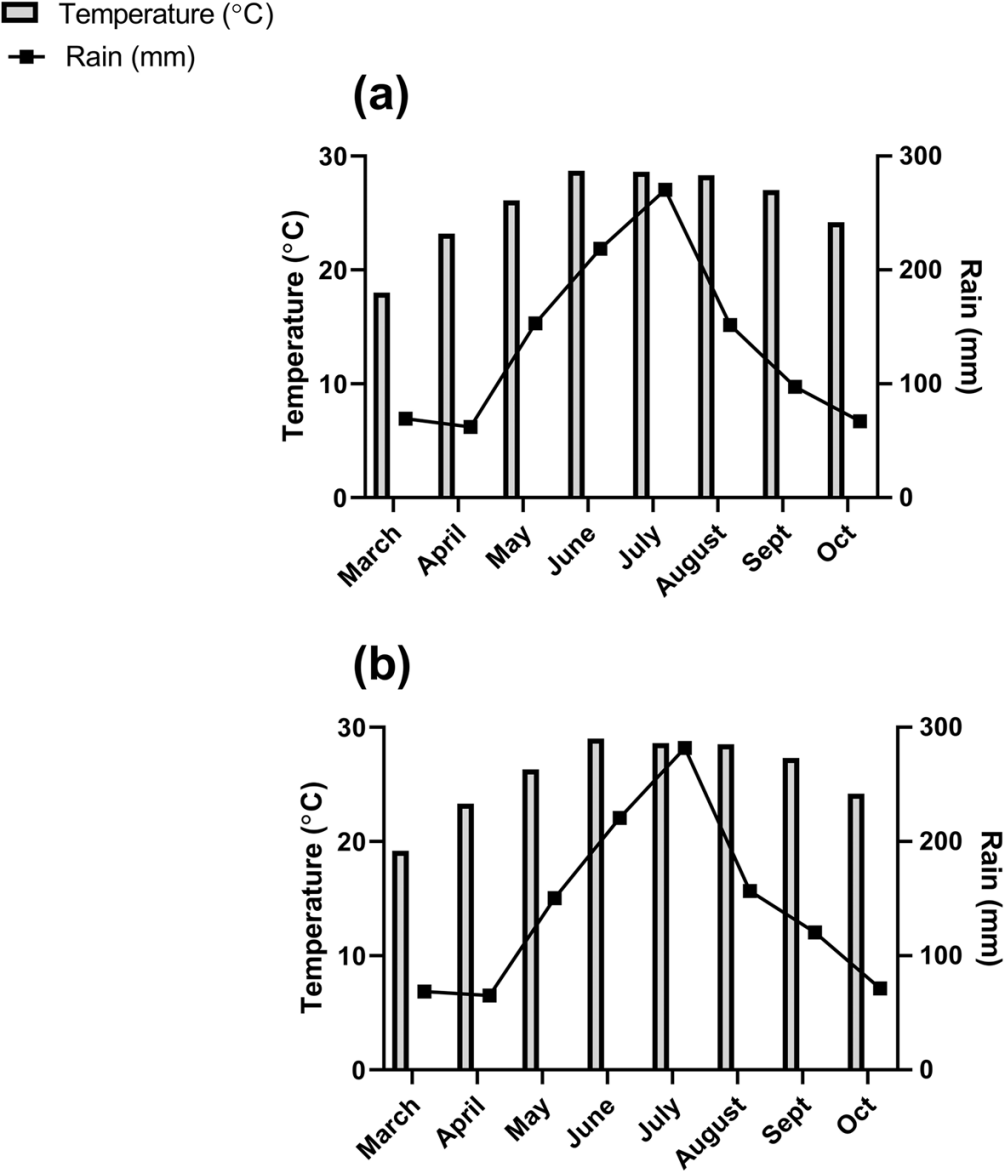
Weather forecast report of the experimental site. **a** and **b** represent the recorded temperature (°C) and rainfall (mm) of the year 2021 and 2022 respectively during the experiment period (from sowing to harvesting). The bar graph represents temperature and the line graph resepresents rainfall

In the current study, a relay intercropping system of maize and soybean was adopted, where two rows of maize were planted with two rows of soybean on a plot size of 22.5 m^2^ (4.5 m long and 5 m wide). However, for maize and soybean mono-cropping, the plot size was kept at 18 m^2^ (4.5 m long and 4 m wide). The N fertilizers were applied as urea (46% N), whereas for the other fertilizers such as phosphorus as a pentoxide P_2_O_5_ (46% P) and potassium were applied at the rate of 100 kg ha^−1^ each as basal doses. Maize and soybean planting was done at an inter-plant distance of 30 cm and 20 cm with a density of 60,000 maize plant ha^−1^ and 100,000 soybean plants ha^−1^, respectively in both mono-cropping and intercropping. However, the distance between rows was kept at 40 cm in the mono-cropping system for both maize and soybean, whereas the distance between rows for these crops under the intercropping system was 60 cm (Fig. 1). The maize was planted on March 15, 2021, and harvested on August 10, 2021, whereas for the soybean, planting was carried out on June 10, 2021 with harvesting taking place on October 20, 2021. In 2022, for maize, planting took place on March 10, 2022, and harvesting on August 08, 2022 whereas the respective dates for soybean were June 08 and October 15. Throughout the growth stages, plants were routinely irrigated to maintain the soil at 60–70% of its field water-holding capacity. A small hand spade was used to remove weeds, while plants were sprayed with pesticides and fungicicides to reduce the attack by pests and diseases, respectively. Metrological data (i.e., temperature and rainfall) were meticulously observed and recorded during the experiment period (Fig. 2).

### Data collection

#### Grain yield, biomass dry matter, and 1000-grain weight

Maize and soybean plants were harvested at full maturity, followed by threshing of the cobs for maize and pods for soybean. The grain yield and 1000-grain weight were then determined. Following threshing, a sample of about 1 kg per treatment of the residual plant straw was taken and oven-dried for 72 h at 65 degrees Celsius to a consistent weight for dry matter biomass calculation.

### LER and LER_N_

The land equivalent ratio (LER) and land equivalent ratio for N fertilization (LER_N_) were computed as indicated in Eqs. 1 and 2 [35].

1 $$\text{L}\text{E}\text{R}=\left(\frac{{\text{Y}}_{\text{i}\text{m}}}{{\text{Y}}_{\text{m}\text{m}}}+\frac{{\text{Y}}_{\text{s}\text{i}}}{{\text{Y}}_{\text{s}\text{m}}}\right)$$

2 $${\text{L}\text{E}\text{R}}_{\text{N}}=\left(\frac{{\text{Y}}_{\text{i}\text{m}\text{N}}}{{\text{Y}}_{\text{m}\text{m}\text{N}}}+\frac{{\text{Y}}_{\text{s}\text{i}\text{N}}}{{\text{Y}}_{\text{s}\text{m}\text{N}}}\right)$$

In terms of LER, Ymm, and Ysm stand for the crop yields under mono-cropping, while Yim and Ysi illustrate the crop yield of maize and soybean crops, respectively when grown in intercropping. Likewise, for LER_N_, the Ymm_N_ and Ysm_N_ represent the grain yield of maize and soybean in their mono-cropping system under N fertilization, and Yim_N_ and Ysi_N_ represent the grain yields of maize and soybean crops respectively grown in intercropping under N fertilization. Generally, LER is used to examine the competitiveness for resources between intercrops, whereas LER_N_ is used to determine the efficient use of applied N fertilizer. Whenever LER and LER_N_ are equal to 1, it means intercropping and mono-cropping systems equally utilize the resources and N fertilizers. This implies that the greater the variables above 1, means intercropping has a complementary effect and better use of the applied N fertilizer. Other cintrary, a value of less than 1, means interspecific competition is occuring between intercrops for resources and applied nutrients. So, the higher the LER and LER_N_, the greater the benefit of intercropping, efficient the use of the applied fertilizers, and vice versa [36].

### Plant nutrient content

After harvesting at full maturity, the plant samples were dried, crushed, and sieved with a 2 mm sieve to collect fine powder to determine plant nutrient content. Plant macronutrients such as N, P & K were determined following the standard protocol of wet digestion. In brief, plant samples were digested in a 2:1 solution of sulfuric acid (H_2_SO_4_) and hydrogen peroxide (H_2_O_2_). Different apparatus were used to determine these macronutrients according to previously standard procedures. For example, the Kjeldahl apparatus was used to determine plant nitrogen (N) content [37], a colorimeter was used to test phosphorus (P) in plants [38], and a flame photometer was used for potassium (K) content in plant samples (Hitachi Z-2000, Tokyo). The dry ashing method was used to evaluate plant micronutrients (e.g., Ca, Zn, Fe, Mn, and Mg). This process involved the ashing of plant materials for six hours at 550 °C, adding 5 mL of hydrochloric acid, and then gradually topping with distilled water to make a 25 mL solution. Plastic vials were used to retain the slurry after it had been filtered through the Whatman No. 5 filter paper. The standard curve of the atomic absorption spectrophotometer was used to determine the micronutrient content values (Hitachi Z-2000, Tokyo).

#### Soil physio-chemical characteristics

Soil samples were drawn from each treatment plot at a depth of 0–20 cm to examine the various soil physio-chemical parameters. For better results, all samples were air-dried, powdered, and passed through 2 mm sieves. Soil pH was tested by pH meter from homogeneous soil-water solution extraction [39]. SOM (soil organic matter) was determined by acid digestion procedure [40]. AN (available nitrogen) using the alkali hydrolytic diffusion producer [41]. NH_4_ and NO_3_ by 2 mol·L^−1^ KCl extraction with a flow analyzer, and available P using the sodium-bicarbonate (NaHCO_3_) extraction method [42]. AK (soil available K) was estimated by extracting the samples with 1 M ammonium acetate (NH_4_Ac) and measuring the element with a flame photometer (Hitachi Z-2000, Tokyo).

#### Soil enzymatic analysis

The protease (PT) activity was measured by the ninhydrin colorimetry method. In brief, 1 g of soil was cultured in ninhydrin for 24 h at 37 °C, and PT activity was measured and expressed in milligrams of amino nitrogen [43]. For sucrase (SC) activity, 5 g of air-dried soil was incubated with 15 mL of sucrase. After incubation, the reaction solution was filtered as quickly as possible through the quantitative filter paper using five microliters (5 mL) of phosphate buffer (pH 5.5) and five drops of toluene. Filtrate (1 mL) was combined with 3 mL salicylic acid and heated in a water bath to 100 °C for 5 min before being adjusted to 50 mL and cooled with deionized water. This was followed by measurement of the SA activity through spectrophotometry at 508 nm [44]. For urease (UE) activity, five grams of soil were incubated with 10 mL of citrate phosphate buffer (pH 6.7) and 5 mL of a 10% urea solution at 38 °C for three hours to determine the amount of urease activity. Using a spectrophotometer set at 578 nm, the released NH_4_^+^ was measured to assess UE activity [45]. Additionally, nitrophenyl phosphate disodium (PhOH mg g^1^, 37 _C, 24 h) and KMnO_4_ (0.1 mol L1 KMnO_4_ ug g^1^, 30 °C, 20 h) were used to measure the activity of acid phosphatase (AP) and catalase (CT), respectively [46].

### Statistical analysis

Data were collected, gathered, and arranged in Ms excel. The data were statistically analyzed separately for maize and soybean crops by factorial ANOVA test using MS statistix 8.1 software. Graphical representation of the data was made by GraphPad Prism 9.1 software. The comparison between mono-cropping and intercropping under different fertilizations for maize and soybean crops was made discretely. For example, maize/soybean intercropping with or with absence of N fertilization, comparison was made with the corresponding mono-cropping (i.e., N_0_MM/N_0_SM, N_1_MM/N_1_SM vs. N_0_MI/N_0_SI, N_1_MI/N_1_SI). The means of the treatments were compared using a split-plot design with two-factor factorial analysis, with nitrogen levels being taken as the main effect and planting pattern as sub-effect at *p* ≤ 0.05 level of significance (LSD test).

## Results

### Yield and yield indices

**Fig. 3:**
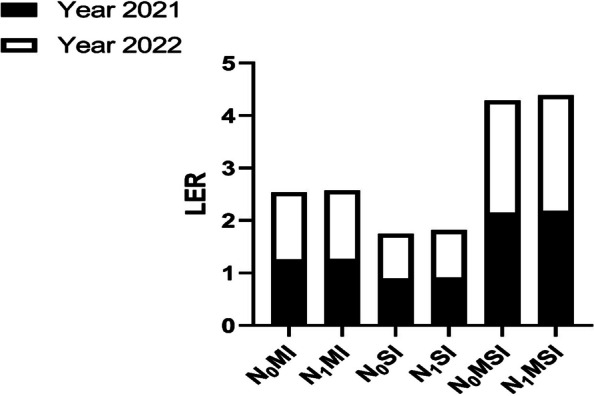
LER and LER_N_ in the year 2021 and 2022. N_0_M1; Maize intercropping without N fertilization, N_1_MI; Maize intercropping under N fertilization, N_0_SI; Soybean intercropping without N fertilization, N_1_SI; Soybean intercropping under N fertilization, N_0_MSI; Maize-Soybean intercropping without N fertilization, N_1_MSI; Maize-Soybean intercropping under N fertilization

Intercropping had a significant (*p* ≤ 0.05) effect on the yield and yield indices of maize and soybean crops. With absence of N or with its presence, intercropping enhanced the yield indices of maize crops but reduced that of soybean crops (Tables 1 and 2). In 2021, when compared with mono-cropping treatments (i.e., N_0_MM and N_1_MM), N_1_MI had the maximum grain yield (10,105 kg ha^−1^), biomass dry matter (13,893 kg ha^−1^) and 1000-grain (420 g) weight of maize crop followed by N_0_MI, which produced the grain yield (9103 kg ha^−1^), biomass dry matter (11,478 kg ha^−1^) and 1000-grain (357 g) weight of maize crop. Similarly, in 2022, the maximum grain yield (11,705 kg ha^−1^), biomass dry matter (14,093 kg ha^−1^) and 1000-grain (449 g) weight of maize crop was recorded in N_1_MI followed by N_0_MI, which had grain yield of 9493 kg ha^−1^, biomass dry matter of 12,103 kg ha^−1^ and 1000-grain of 382 g weight of maize crop when compared with mono-cropping treatments (i.e., N_0_MM and N_1_MM). In contrast, these indices of the soybean crop were higher under mono-cropping (i.e., N_0_SM and N_1_SM) than in intercropping treatments (i.e., N_0_SI and N_1_SI). For instance, the maximum grain yield (9723 and 9948 kg ha^−1^), biomass dry matter (11,984 and 12,634 kg ha^−1^) and 1000-grain weight (193 and 198 g) of soybean crop was recorded in N_1_SM followed by N_0_SM, whose grain yield was 8946 vs. 9370.40 kg ha^−1^, with biomass dry matter of 11,844 vs. 11,255 kg ha^−1^ and 1000-grain weight of 183 vs. 185 g in the year 2021 and 2022, respectively. Additionally, the LER and LER_N_ values were always greater than 1 in the intercropping system, suggesting yield advantages of intercropping and better use of applied N fertilizer (Fig. 3).

**Table 1:** Yield and yield indices of maize and soybean under maize-soybean intercropping and N fertilization in the year 2021

Treatment		Grain yield (kg ha^−1^)	Biomass dry matter (kg ha^−1^)	1000-grain weight (g)
Maize crop				
N_0_MM		7273.03 ± 342.6 c	10368.45 ± 536.2 b	305.53 ± 30.3 c
N_0_MI		9103.42 ± 639.1 b	11477.90 ± 691.1 b	356.98 ± 33.7 b
N_1_MM		7959.14 ± 104.9 c	11220.40 ± 362.1 b	344.59 ± 32.1 bc
N_1_MI		10105.26 ± 497.1 a	13893.12 ± 803.8 a	420.37 ± 22.8 a
Significance	NL	0.029*	0.031*	0.011**
	PP	0.000***	0.003***	0.003**
	NL*PP	0.608ns	0.100ns	0.397ns
Soybean crop				
N_0_SM		8945.39 ± 379.5 ab	11844.20 ± 438.7 b	182.67 ± 8.1 b
N_0_SI		8012.84 ± 530.9 c	9672.75 ± 489.5 c	167.78 ± 5.5 c
N_1_SM		9722.86 ± 519.2 a	11983.91 ± 225.5 a	192.47 ± 3.8 a
N_1_SI		8885.42 ± 77.06 b	10762.76 ± 876.7 ab	180.96 ± 7.9 b
Significance	NL	0.041*	0.032*	0.006**
	PP	0.000***	0.003**	0.000***
	NL*PP	0.742ns	0.646ns	0.432ns

The mean values with SD (*n* = 4) are shown in the Table

The mean with similar lowercase letters down the column are significantly different from each other at the LSD test *P* ≤ 0.05 level of probability. N_0_: 0 kg N ha^−1^, N_1_: 225 kg N ha^-1^ for maize and 100 kg N ha^-1^ for soybean

*NL* Nitrogen levels, *PP* Planting pattern

*p ≤ 0.05

**p ≤ 0.01

**p ≤ 0.0001

ns *p* > 0.05

**Table 2:** Yield and yield indices of maize and soybean under maize-soybean intercropping and N fertilization in the year 2022

Treatment		Grain yield (kg ha^−1^)	Biomass dry matter (kg ha^−1^)	1000-grain weight (g)
Maize crop				
N_0_MM		7428.05 ± 252.9 c	10568.45 ± 831.4 c	318.37 ± 34.7 c
N_0_MI		9493.40 ± 751.2 b	12103.10 ± 494.6 b	381.72 ± 37.2 b
N_1_MM		8984.15 ± 230.2 b	11770.43 ± 597.2 bc	358.28 ± 34.8 bc
N_1_MI		11705.28 ± 753.7 a	14092.83 ± 1167.9 a	448.81 ± 26.5 a
	NL	0.004**	0.038*	0.005**
Significance	PP	0.001**	0.002**	0.002**
	NL*PP	0.434ns	0.352ns	0.411ns
Soybean crop				
N_0_SM		9370.40 ± 615.7 b	11255.21 ± 766.7 b	184.95 ± 5.6 b
N_0_SI		7962.81 ± 387.51 c	9973.15 ± 600.9 c	174.11 ± 4.6 c
N_1_SM		9947.87 ± 466.7 a	12633.91 ± 422.5 a	197.78 ± 7.5 a
N_1_SI		8950.42 ± 429.6 ab	11612.76 ± 955.5 b	189.98 ± 7.1 b
	NL	0.020*	0.014*	0.002**
Significance	PP	0.005**	0.001**	0.000***
	NL*PP	0.497ns	0.573ns	0.221ns

The mean values with SD (*n* = 4) are shown in the Table

The mean with similar lowercase letters down the column are significantly different from each other at the LSD test *P* ≤ 0.05 level of probability. N_0_: 0 kg N ha^−1^, N_1_: 225 kg N ha^-1^ for maize and 100 kg N ha^-1^ for soybean

*NL *Nitrogen levels, *PP* Planting pattern

*p ≤ 0.05

**p ≤ 0.01

***p ≤ 0.0001

ns *p* > 0.05

### N, P, K, and Ca content of maize and soybean

**Fig. 4:**
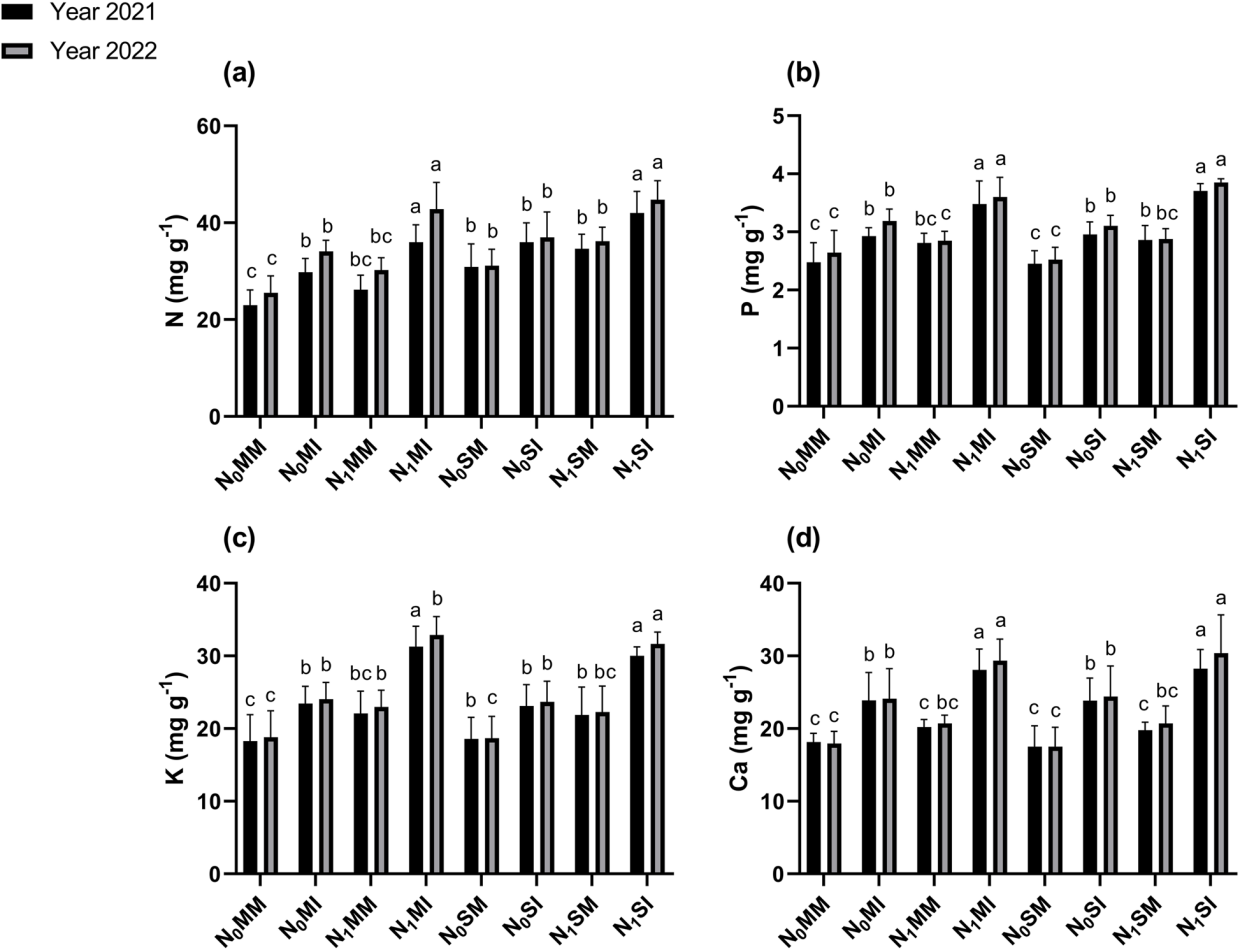
Plant Nutrients such as N; (**a**), P; (**b**), K; (**c**), and Ca; (**d**) of maize and soybean crops under different planting patterns and N fertilization in the years 2021 and 2022. N_0_MM; Maize mono-cropping without N fertilization, N_0_MI; Maize intercropping without N fertilization, N_1_MM; Maize mono-cropping under N fertilization, N_1_MI; Maize intercropping under N fertilization, N_0_SM; Soybean mono-cropping without N fertilization, N_0_SI; Soybean intercropping without N fertilization, N_1_SM; Soybean mono-cropping under N fertilization, N_1_SI; Soybean intercropping under N fertilization. The lowercase letters on the column represent the significant difference among the treatments at LSD (*p* ≤ 0.05) level of probability

When compared with mono-cropping treatments, intercropping treatments either with N or with absence of N fertilization significantly (*p* ≤ 0.05) increased the N, P, K, and Ca contents of maize and soybean crops in both years (Fig. 4). In 2021, the P, N, K, and Ca content of maize crops increased by 18, 21, 28, and 31% respectively, under N_0_MI treatment, and further increased by 24,25, 42, and 39%, respectively under N_1_MI treatment when compared with mono-cropping treatments (i.e., N_0_MM and N_1_MM). However, the N, P, K, and Ca content of soybean increased by 21, 16, 24, and 36%, respectively under N_0_SI, and further increased by 30, 21, 37, and 43% under N_1_SI treatment as compared with the mono-cropping treatments (i.e., N_0_SM and N_1_SM). Similarly, in 2022, when compared with mono-cropping treatment (i.e., N_0_SM and N_1_SM), intercropping resulted in increased N, P, K, and Ca content in maize crop by 21, 23, 28, and 34% respectively, in N_0_MI treatment, and by 27, 29, 43, and 42%, respectively in N_1_MI treatment. For soybean , N, P, K, and Ca content increased by 23, 19, 27, and 40%, respectively under N_0_SI, with auxiliary increases of 34, 24, 42, and 47% being noted under N_1_SI treatment when compared with mono-cropping treatment (i.e., N_0_SM and N_1_SM).

### Mg, Fe, Mn, and Zn content of maize and soybean

**Fig. 5:**
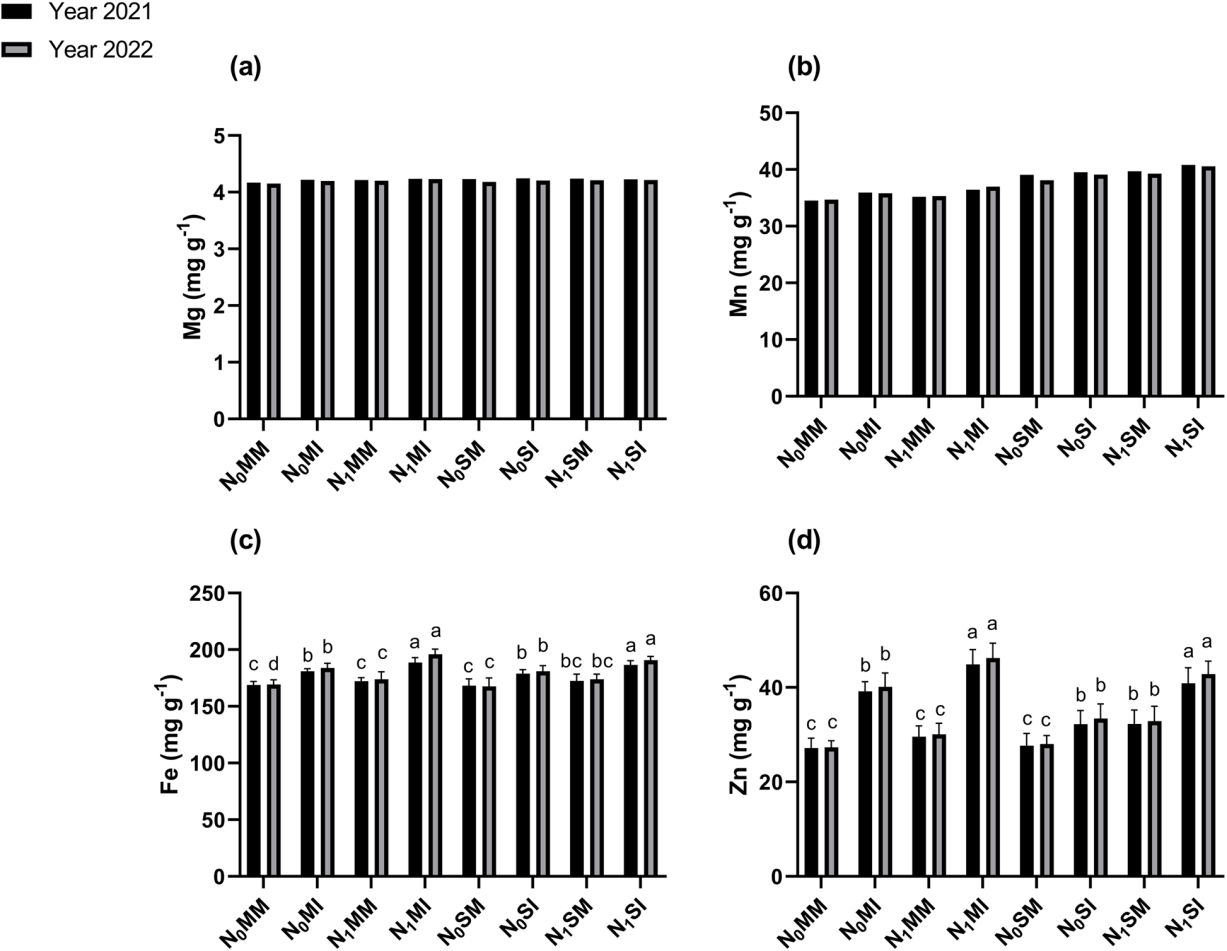
Plant Nutrients such as Mg; (**a**), Mn; (**b**), Fe; (**c**) and Zn; (**d**) of maize and soybean crops under different planting patterns and N fertilization in the years 2021 and 2022. N_0_MM; Maize mono-cropping without N fertilization, N_0_MI; Maize intercropping without N fertilization, N_1_MM; Maize mono-cropping under N fertilization, N_1_MI; Maize intercropping under N fertilization, N_0_SM; Soybean mono-cropping without N fertilization, N_0_SI; Soybean intercropping without N fertilization, N_1_SM; Soybean mono-cropping under N fertilization, N_1_SI; Soybean intercropping under N fertilization. The lowercase letters on the column represent the significant difference among the treatments at LSD (*p* ≤ 0.05) level of probability

Intercropping either with absence of N or with N fertilization significantly (*p* ≤ 0.05) affected Fe and Zn contents of maize and soybean crops, nevertheless, the Mg and Mn content did not show any significant differences when compared with their respective mono-cropping system (Fig. 5). For instance, in the year 2021, when compared with mono-cropping treatment (i.e., N_0_MM and N_1_MM), the maize crop under intercropping treatments such as N_0_MI and N_1_MI had higher Fe content by 7 and 44% and Zn content by 10% and 52%, respectively. Similarly, in 2022, when compared with mono-cropping system the Fe and Zn contents of maize crops increased by 9 and 47%, and by 13% and 54%, in the N_0_MI and N_1_MI intercropping treatments respectively. Similar patterns were equally noticed for soybean crops in both years. For example, in 2021, the Fe and Zn content of soybean increased by 6 and 16%, respectively in N_0_SI intercropping treatment with further increases of 8 and 27%, respectively being noted in N_1_MI intercropping treatment as compared with the mono-cropping treatments (i.e., N_0_SM and N_1_SM). Likewise, in 2022, the Fe and Zn content of soybeans was higher by 8 and 19%, respectively, in N_0_SI treatment. Nevertheless, these nutrient contents (for soybean) were observed to have increased further by 10 and 30%, respectively, in N_1_MI treatment as compared with the mono-cropping treatments (i.e., N_0_SM and N_1_SM). Overall, when compared with mono-cropping treatments, intercropping with or with absence of N fertilization resulted in increased Fe and Zn content of maize and soybean in both years.

### Soil physio-chemical characteristics

Intercropping with or without N application significantly influenced the physio-chemical properties and nutrient availability in the soil (Tables 3 and 4). Results showed that intercropping treatments had enhanced soil available nutrient as well as organic matter, but reduced soil pH as compared with mono-cropping treatments. However, intercropping treatments under N fertilization showed improvement for such indices. For instance, in 2021, when compared with mono-cropping treatments (i.e., N_0_MM, N_0_SM, N_1_MM, N_1_SM), the maximum soil available N (87.80 mg kg^−1^), available P (81.70 mg kg^−1^), available K (95.36 mg kg^−1^), NH_4_ (2.71 mg kg^−1^), NO_3_ (3.77 mg kg^−1^) and organic matter (20.84 mg kg^−1^) were recorded in N_1_MSI intercropping treatment followed by N_0_MSI intercropping treatment, which had lower levels of soil available N (79.70 mg kg^−1^), available P (77.71 mg kg^−1^), available K (86.86 mg kg^−1^), NH_4_ (1.80 mg kg^−1^), NO_3_ (2.99 mg kg^−1^) and organic matter (17.9 mg kg^−1^). Similarly, in 2022, intercropping treatments such as N_1_MSI had higher soil available N (91.55 mg kg^−1^), available P (83.85 mg kg^−1^), available K (97.81 mg kg^−1^), NH_4_ (2.81 mg kg^−1^), NO_3_ (3.86 mg kg^−1^) and organic matter (22.09 mg kg^−1^) relative to N_0_MSI treatment, with respective values of 82.70, 79.88, 89.86, 1.88, 3.09, and 18.50 mg kg^−1^, as compared with mono-cropping treatments (i.e., N_0_MM, N_0_SM, N_1_MM, N_1_SM). In contrast, when compared with mono-cropping treatments, intercropping treatments, had lower soil pH levels. For example, N_0_MSI and N_1_MSI had low soil pH of 5.80 and 5.91, respectively in 2021, and 5.67 and 5.75, in the respective treatments in 2022.

**Table 3:** Soil physio-chemical characteristics as influenced by different planting patterns and N fertilization in the year 2021

Treatment		AN (mg kg^−1^)	AP (mg kg^−1^)	AK (mg kg^−1^)	OM (g kg^−1^)	NH_4_ (mg kg^−1^)	NO_3_ (mg kg^−1^)	pH
N_0_MM		68.13 ± 4.8 cd	73.12 ± 3.9 b	76.72 ± 5.3 c	14.09 ± 2.5 c	1.58 ± 0.1 c	2.68 ± 0.2 d	6.41 ± 0.2 b
N_0_SM		73.62 ± 3.5 cd	72.63 ± 4.9 b	81.56 ± 5.9 bc	14.47 ± 2.9 c	1.69 ± 0.1 b	2.80 ± 0.1 d	6.45 ± 0.3 ab
N_0_MSI		79.70 ± 2.8 b	77.71 ± 1.8 ab	86.86 ± 4.6 ab	17.59 ± 1.5 b	1.80 ± 0.2 bc	2.99 ± 0.1 c	5.80 ± 0.1 c
N_1_MM		72.08 ± 5.1 de	74.98 ± 4.2 ab	81.41 ± 5.8 bc	15.86 ± 2.3 bc	2.21 ± 0.1 b	3.26 ± 0.1 b	6.56 ± 0.1 ab
N_1_SM		76.79 ± 2.5 bc	74.30 ± 4.4 b	84.60 ± 5.8 bc	16.52 ± 2.6 bc	2.41 ± 0.1 a	3.33 ± 0.1 b	6.68 ± 0.1 a
N_1_MSI		87.80 ± 3.4 a	81.70 ± 3.1 a	95.36 ± 2.3 a	20.84 ± 1.3 a	2.71 ± 0.3 a	3.77 ± 0.3 a	5.91 ± 0.5 c
Significance	NL	0.009**	0.019**	0.043*	0.021*	0.000***	0.002**	0.03*
	PP	0.000***	0.028*	0.000***	0.000***	0.000***	0.000***	0.000***
	NL*PP	0.126ns	0.846ns	0.187ns	0.684ns	0.012*	0.044*	0.775ns

The mean values with SD (*n* = 4) are shown in the Table

The mean with similar lowercase letters down the column are significantly different from each other at the LSD test *p* ≤ 0.05 level of probability

N_0_: 0 kg N ha^−1^, N_1_: 225 kg N ha^-1^ for maize and 100 kg N ha^-1^ for soybean

*NL* Nitrogen levels

*PP* Planting pattern

*p ≤ 0.05

**p ≤ 0.01

***p ≤ 0.0001

ns *p* > 0.05

**Table 4:** Soil physio-chemical characteristics as influenced by different planting patterns and N fertilization in the year 2022

Treatment		AN (mg kg^−1^)	AP (mg kg^−1^)	AK (mg kg^−1^)	OM (g kg^−1^)	NH_4_ (mg kg^−1^)	NO_3_ (mg kg^−1^)	pH
N_0_MM		65.38 ± 3.7 d	73.96 ± 4.2 b	77.22 ± 4.1 d	14.60 ± 2.0 c	1.61 ± 0.1 f	2.71 ± 0.2 c	6.43 ± 0.2 c
N_0_SM		72.61 ± 2.1 cd	73.35 ± 4.9 b	80.06 ± 6.6 cd	14.73 ± 2.1 c	1.73 ± 0.1 e	2.82 ± 0.2 c	6.53 ± 0.3 b
N_0_MSI		82.70 ± 3.7 b	79.88 ± 1.2 ab	89.86 ± 3.9 b	18.50 ± 1.1 b	1.88 ± 0.2 d	3.09 ± 0.4 b	5.67 ± 0.3 bc
N_1_MM		70.83 ± 5.3 cd	75.62 ± 3.9 b	81.66 ± 4.5 cd	16.36 ± 2.5 bc	2.25 ± 0.1 c	3.27 ± 0.1 b	6.61 ± 0.2 b
N_1_SM		73.79 ± 3.3 c	74.67 ± 4.4 b	85.10 ± 4.1 bc	17.02 ± 2.1 bc	2.45 ± 0.1 b	3.34 ± 0.1 b	6.76 ± 0.1 a
N_1_MSI		91.55 ± 3.8 a	83.86 ± 2.3 a	97.81 ± 1.3 a	22.09 ± 1.1 a	2.81 ± 0.3 a	3.86 ± 0.3 a	5.75 ± 0.3 a
Significance	NL	0.010*	0.015*	0.009**	0.033*	0.000***	0.004**	0.023*
	PP	0.000***	0.006**	0.000***	0.000***	0.000***	0.000***	0.000***
	NL*PP	0.297ns	0.808ns	0.557ns	0.465ns	0.001**	0.058ns	0.684ns

The mean values with SD (*n* = 4) are shown in the Table

The mean with similar lowercase letters dowm the column are significantly different from each other at the LSD test *p* ≤ 0.05 level of probability

N_0_: 0 kg N ha^−1^, N_1_: 225 kg N ha^-1^ for maize and 100 kg N ha^-1^ for soybean

*NL* Nitrogen levels, *PP* Planting pattern

*p ≤ 0.05

**p ≤ 0.01

***p ≤ 0.0001

ns *p* > 0.05

### Soil enzymatic activities

**Fig. 6:**
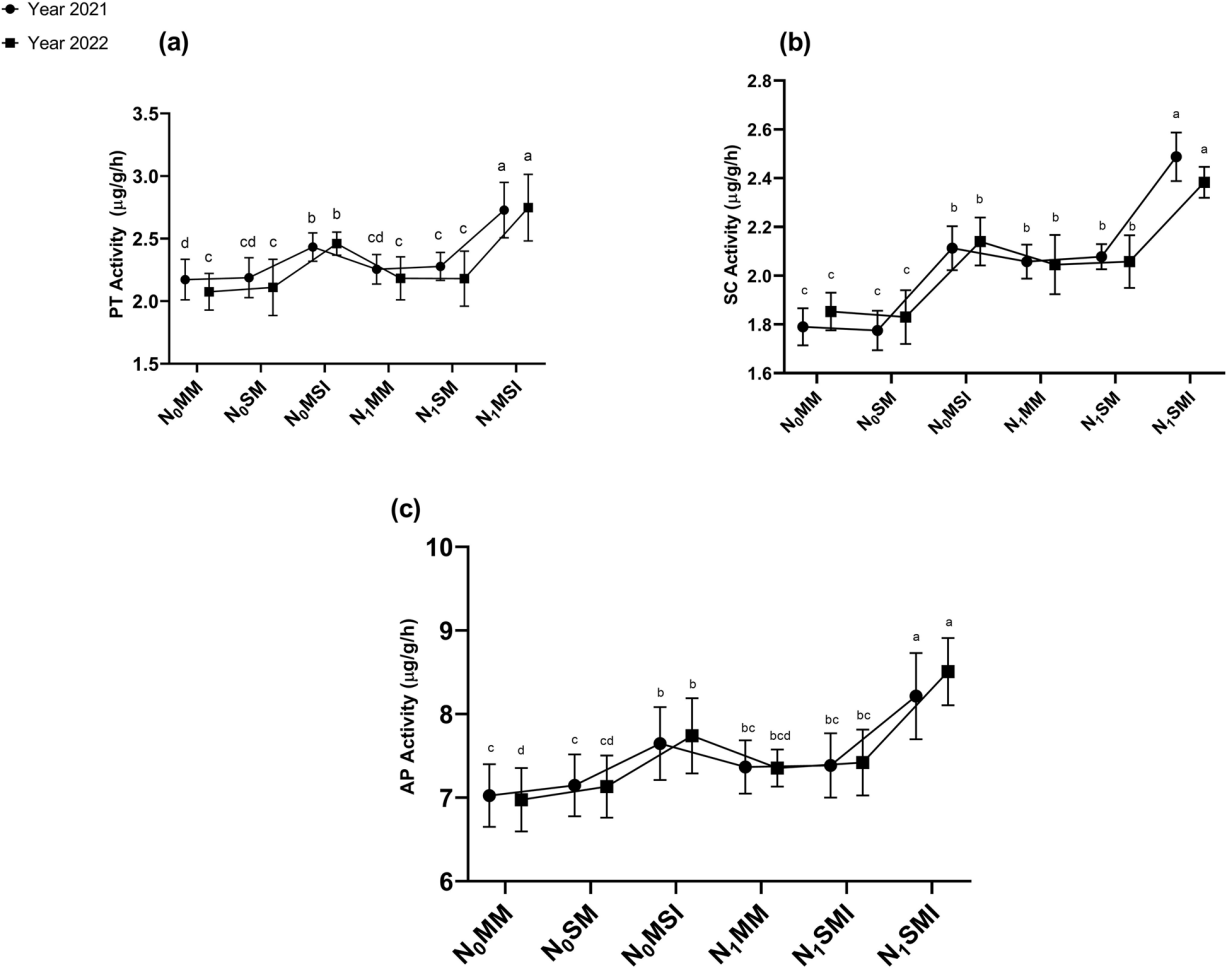
Soil enzyme activities under different planting patterns and N fertilization in the years 2021 and 2022. **a** Protease Activity (PT), **b** Sucrase Activity (SC), **c** Acid Phosphatase Activity (AP). N_0_MM; Maize mono-cropping without N fertilization, N_0_SM; Soybean mono-cropping without N fertilization, N_0_MSI; Maize-Soybean intercropping without N fertilization, N_1_MM; Maize mono-cropping under N fertilization, N_1_SM; Soybean mono-cropping under N fertilization N_1_MSI; Maize-soybean intercropping under N fertilization. The lowercase letters on the column represent the significant difference among the treatments at LSD (*p* ≤ 0.05) level of probability

**Fig. 7:**
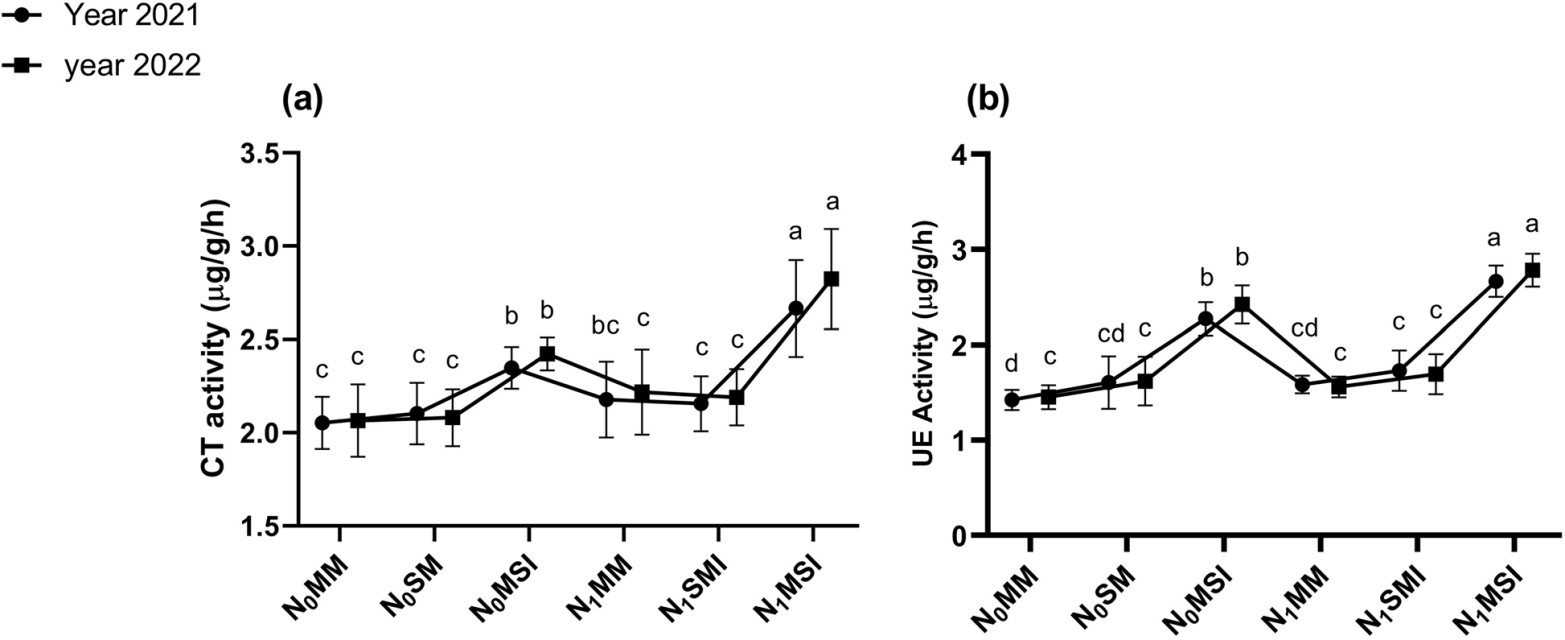
Soil enzyme activities under different planting patterns and N fertilization in the years 2021 and 2022. **a** Catalase Activity (CT), **b** Urease Activity (UE). N_0_MM; Maize mono-cropping without N fertilization, N_0_SM; Soybean mono-cropping without N fertilization, N_0_MSI; Maize-Soybean intercropping without N fertilization, N_1_MM; Maize mono-cropping under N fertilization, N_1_SM; Soybean mono-cropping under N fertilization N_1_MSI; Maize-soybean intercropping under N fertilization. The lowercase letters on the column represent the significant difference among the treatments at LSD (*p* ≤ 0.05) level of probability

Compared with mono-cropping, intercropping treatments with/with absence of N fertilizer application significantly (*p* ≤ 0.05) altered the soil enzymatic activities (Figs. 6 and 7). The intercropping treatments with or with absence of N fertilizers application enhanced the soil enzymatic activities, but these enzyme activities were more pronounced in the N-fertilized intercropping treatments as compared with mon-cropping. In 2021, the soil PT, SC, AP, UE, and CT activity increased by 12, 10, 9, 60, and 14% in the N_0_MSI intercropping treatment, and further increased by 21, 13, 12, 68, and 22% in the N_1_MSI intercropping treatment as compared with mono-cropping treatments (i.e., N_0_MM, N_0_SM, N_1_MM, and N_1_SM). On the other hand, in 2022, the PT, SC, AP, UE, and CT activity increased by 19, 16, 11, 67, and 17% receptively in N_0_MSI, and further increased by 26, 17, 16, 77, and 29% respectively in N_1_MSI intercropping treatments compared with their mono-cropping treatments (i.e., N_0_MM, N_0_SM, N_1_MM, and N_1_SM).

### Correlation analysis

**Fig. 8:**
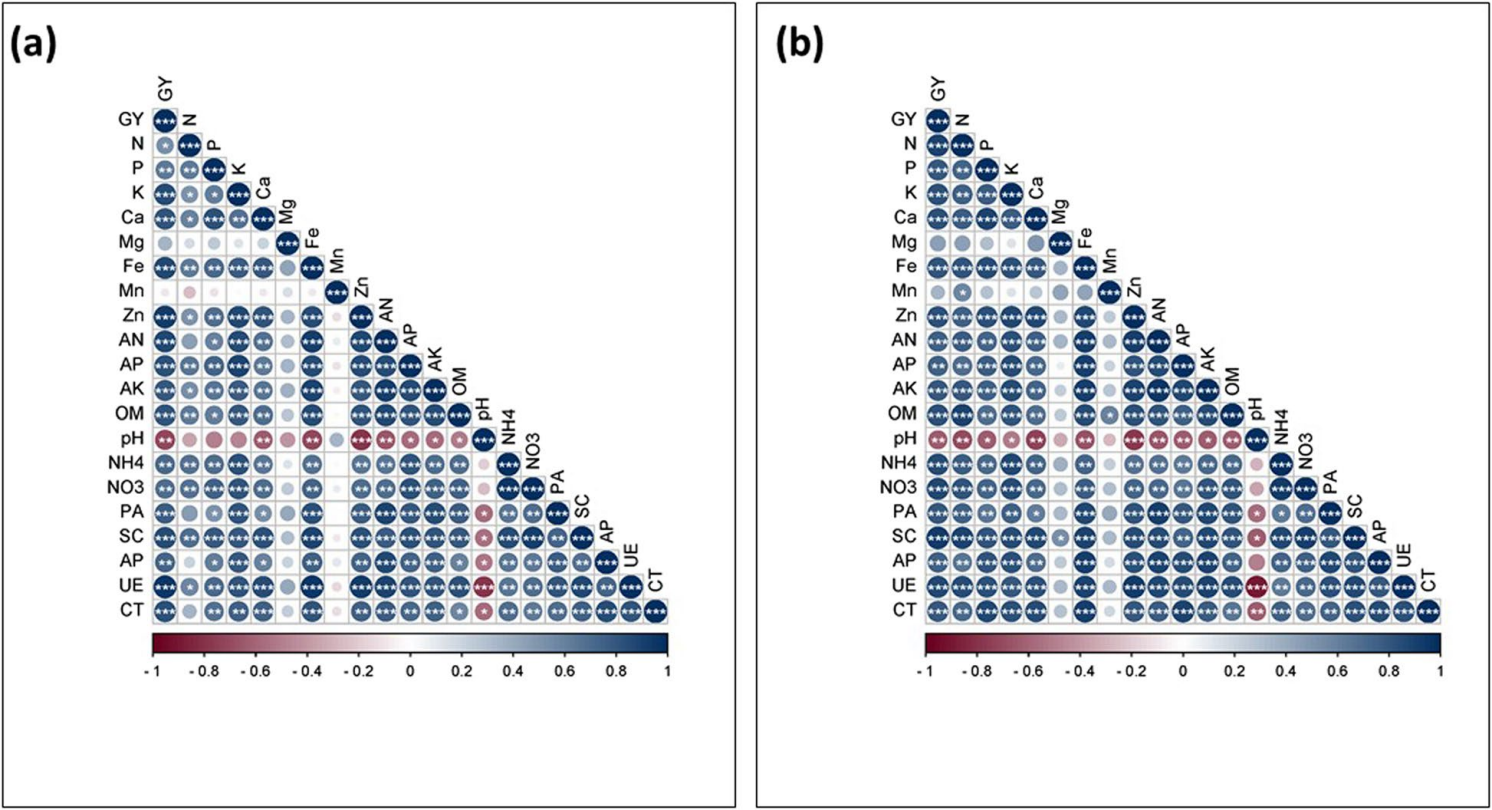
Relationship of maize yield with plant nutrients, soil physio-chemical characteristics and soil enzymes of the year 2021 (**a**) and 2022 (**b**). The color gradients represent the positive and negative relationship, while the * indicates the significance level at LSD (*p* ≤ 0.05) level of probability

**Fig. 9:**
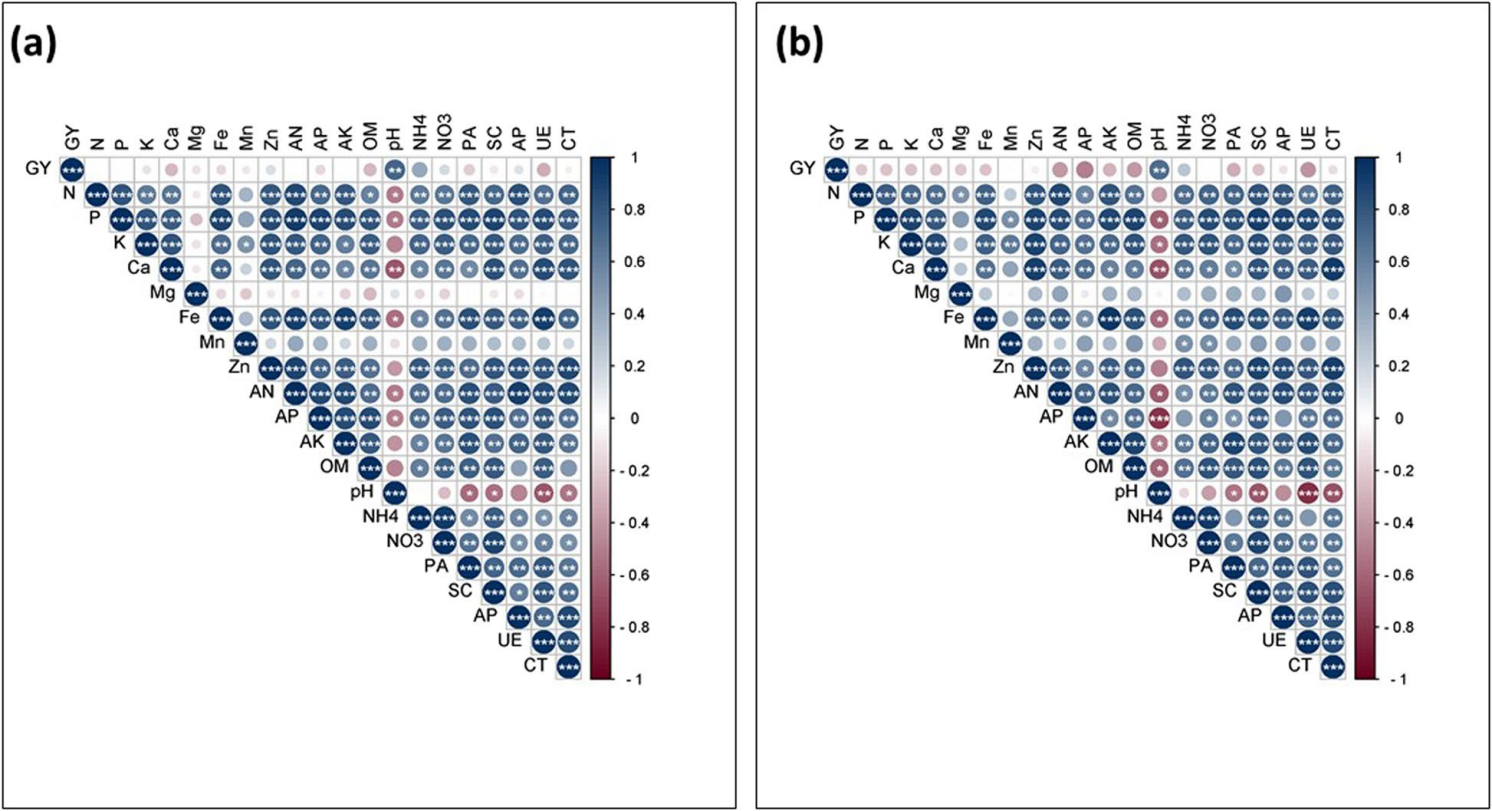
Relationship of soybean yield with plant nutrients, soil physio-chemical characteristics and soil enzymes of the year 2021 (**a**) and 2022 (**b**). The color gradients represent the positive and negative relationship, while the * indicates the significance level at LSD (*p* ≤ 0.05) level of probability

The correlation analysis was used to determine the relationship of grain yield of maize and soybean crops with plant nutrient content, soil physio-chemical characteristics and soil enzymes. The correlation result of the maize crop showed that the grain yield of the maize crop was significantly positively correlated with the majority of the plant nutrients, soil physio-chemical characteristics and soil enzymes. However, significantly negatively correlated with soil pH (Fig. 8a and b). In contrast, the grain yield of soybean was negatively correlated with the plant nutrients, soil physio-chemical characteristics and soil enzymes, however, it significantly positively correlated with soil pH (Fig. 9a and b).

## Discussion

### Yield and biomass dry matter

Cereal-legume intercropping is practiced globally to increase crop productivity, better utilize existing natural resources, reduce the use of chemical fertilizers, and establish a sustainable and ecologically friendly agricultural production system. Owing to this, we intercropped maize with soybean with and with absence of N fertilizers application (i.e., N_0_; 0 kg N ha^−1^ and N_1_; 225 N kg ha^−1^ for maize and 100 N kg ha^−1^ for soybean) to increase the nutrient uptake, yield of the crops, and soil fertility. It is undeniable that cereal-legume intercropping, as opposed to mono-cropping, considerably increased crop productivity, owing to the efficient use of available natural resources such as light, water, land, and nutrients [8, 14, 29]. The current study found that maize/soybean intercropping with adequate N fertilization boosted maize crop yield production, biomass dry matter, and 1000-grain weight, but decreased soybean crop yield. Possibly, this could be due the fact that soybean being a legume crop is known for its ability to fix atmospheric N, which helps improve the soil fertility and soil health by enriching it with nutrients, whereas maize as a cereal crop required more nutrients for its normal growth and development, thus intercropping soybean with maize can helps provide more space and more nutrient for its companion maize crop for its normal growth, thereby enhancing its yield [47–50].

Maize and soybean often have different root architectures and depths, with maize typically having a shallower root system, while soybean has deeper taproots. This reduces competition for water and nutrients, as they extract resources from different soil layers, which modifies the underlying rhizosphere environment and improves the soil nutrient availability. Consequently, both crops can access the required nutrients and moisture more efficiently [51]. By planting such different crop types together, resource utilization is more efficient, which can optimize light interception, leading to improved photosynthesis and overall crop growth, which often converts into better yields [48, 52].

Below-ground complementarity, nutrient sharing via interspecific root interactions, and rhizosphere modification in maize-soybean intercropping could also impact crop growth, development, and yield, either directly or indirectly [26, 53]. Adequate N fertilization could further increase the yield of crops under such intercropping system where N is a primary element required for plant growth and development. For example, the element is a key component of amino acids [34], involved in the chlorophyll and photosynthesis, and transfer of energy within plant cells [54], a key constituent of adenosine triphosphate (ATP) and nucleic acids, such as DNA and RNA [55]. It also plays a fundamental role in not only the uptake but also transport of other vital nutrients, such as potassium and phosphorus [27, 56].

Conversely, the lower soybean production in maize/soybean intercropping was due to the shadowing effect of maize, which inhibits the growth of soybean, lowering its yield [57, 58]. Also, being a cereal crop, maize demands more nutrients and other resources (i.e., light and water), which outcompete its companion soybean crop, thereby adversely affecting its growth and yield [18, 20]. Nevertheless, effective N fertilization could mitigate the detrimental effects of maize/soybean intercropping since N aids in lowering underlying nutrient competition between intercrops [20, 59]. Although the current maize/soybean intercropping lowered soybean crop production relative to mono-cropping, such reduction was less in the N-fertilized intercropping. Existing intercropping research, including ours, has shown that intercropping improves crop growth and production compared with mono-cropping systems, which was mostly attributable to the efficient use of accessible natural resources such as water, mineral nutrients, and solar energy [8, 11]. For example, maize-common bean intercropping considerably enhanced the maize crop yield by 12.5% and biomass dry matter (shoots and root) by 31.5% when compared with its mono-cropping system [60]. Several other intercropping studies have shown that the complementarity or facilitative interactions that occur both above and below ground between intercrops consequently improve the soil nutrient availability and increase plant nutrient content and their uptake, which ultimately enhances the crop yield [53, 61, 62], and that confirmed our results.

### Plant nutrients content

The present maize-soybean intercropping has also been shown to have higher plant nutrient contents (i.e., N, P, K, Ca, Fe, and Zn) than in their respective mono-cropping systems, but, such nutrients were more evident in the N fertilized intercropping treatments. Intercropping two different plant species with different rooting architectures will drastically change the underlying soil environment because of their varying rooting behaviours [16, 50]. For example, Cereal and legumes often have different root systems and depths (i.e., shallow and deep), where they exploit resources from different soil layers, which helps both crops access the required nutrients and moisture more efficiently when intercropped together [9, 11]. Such plants with different root behaviours often release different chemicals, causing changes in soil physio-chemical properties and soil enzymes [50], surrounded by different beneficial microorganisms (i.e., Rhizobia, P, and K solubilizing bacteria) for plant/soil nutrient cycling [13]. For instance, legumes can fix atmospheric N in symbiotic relations with rhizobia and enrich the soil with nutrients, which are subsequently available for their companion crop [50]. According to reports, intercropping alfalfa with maize boosted maize crop N uptake by 72% via facilitative root contact, and also improved the soil N availability because of its N fixation ability [20].

Previously, other intercropping studies such as maize-soybean, wheat-maize/soybean (*Glycine max* L), sunflower/sunn hemp, maize/alfalfa, maize/peanut, and wheat/chickpea were used. From all the studies, it is evidently shown that intercropping significantly enhanced plant N uptake, which was attributed to the N fixation ability of legumes, underlying facilitative root interaction/nutrient sharing between intercrops, improved soil nutrient availability, rich availability of soil beneficial microbes and changes occuring in soil rhizosphere because of different chemical released by plant roots [19, 63]. Changes in the underlying soil environment in maize-soybean intercropping may also potentially affect the plant and soil-accessible phosphorous (P), because of the chemical substances (i.e., acid phosphatase and phytase) release by plant root [64], rhizosphere soil acidification as well as a reduction in the soil pH [65], enrichment of P solubilizing bacteria [66], and root exudation [52], which modified the soil rhizosphere, help in P solubilization, mineralization, and mobilization, improve soil Olsen P, and its uptake by plants [9]. For example, the increased P uptake by 20.1% in maize-soybean/peanut intercropping over mono-cropping was mainly because of underlying changes in the soil such as both plant roots and soil microbes release organic acids and extracellular enzymes to activate soil insoluble P and its use efficiency [67].

Additionally, the enhanced level of K and Ca contents in the intercropping system could be owing to cationic antagonism effects between K and Ca [16, 68]. Nonetheless, the increased Fe and Zn concentrations in plant shoots may be related to underlying interspecific processes and changes in rhizospheric soil pH [16, 69]. The increase in Fe and Zn in cereal-legume intercropping is mostly due to ferric reductase activity and rhizosphere acidification by legumes as “Strategy I,” and phytosiderophores root exudation by cereals as “Strategy II.” [68, 70]. These results are supported by several previous intercropping studies including ours, where intercropping was shown to have high plant nutrient content [11, 16, 17].

### Soil physio-chemical charactreistics

Intercropping two different plant species with diverse root behaviors is likely to modify soil physio-chemical characteristics and availability of nutrients [71, 72]. Our maize/soybean intercropping study has considerably changed the soil physio-chemical properties (i.e., pH and organic matter) and available nutrients (i.e., AN, AP, AK, NH_4,_ and NO_3_) as compared with mono-cropping. Several explanations exist that could explain such observations. These incude legumes’ symbiotic N_2_ fixation, which enhances soil health by supplementing it with nutrients [8, 11], root exudations (i.e., sugars, organic, and amino acids and secondary metabolites, such as flavonoids, phenolic, and terpenoids) [73], beneficial soil bacterium (e.g., rhizobia, phosphate-solubilizing, and potassium-solubilizing bacteria) that aids in improving soil physio-chemical properties and nutrient availability [68]. For example, legume crop N fixation ability could help improve soil-accessible N forms (i.e., N, NH_4_, and NO_3_) during intercropping [24]. Changes in soil-accessible phosphorus and potassium in intercropping are most likely due to root-releasing compounds (e.g., acid phosphatases and phytases), changes in the soil pH, and availability of soil bacteria such as P and K solubilizing bacteria [74–76]. Numerous other intercropping investigations have found considerable changes in soil physical and chemical properties and increased soil-accessible nutrients [24, 62, 64, 77], which are consistent with our findings.

### Soil enzymatic activities

Soil enzymes drastically influence biochemical functioning and the overall ecosystem environment of plants and soil [78]. Understanding the enzymatic activity of the soil under various cropping systems will in understanding how intercropping systems might increase soil fertility. According to earlier research, mono-cropping systems may potentially damage the soil’s enzyme system, which would cause a considerable drop in the soil’s enzymatic activity [79]. In our current study, enzyme activities in maize/soybean intercropping systems increased significantly when compared with mono-cropping systems. Among the enzymes, protease is the crucial one that is responsible for the catalysis of N minerals and N cycling [80]. In contrast to mono-cropping, the intercropping system had increased protease activity. This increase in protease activity could be related to a higher soil organic carbon (SOC) content in the topsoil [78]. Sucrase enzymes in the soil hydrolyze sucrase to produce glucose and fructose, and this process is related to the biomass of soil bacteria [78, 81].

According to [65], intercropping can drastically increase soil enzyme activity because of the different rooting systems and the chemicals they produce. Acid phosphatase is the primary enzyme involved in the soil organic P mobilization and also helps in the hydrolysis of ester and phosphoric acid anhydrides [82, 83], transfer esters and anhydrides into phosphate, and accelerate soil P cycling [84]. In our current investigation, acid phosphatase activity was higher in maize/soybean intercropping than in mono-cropping because soybean was found as a species whose roots release a significant amount of acid phosphatase in the soil [11, 85]. Soil urease is another soil enzyme that hydrolyzes urea to produce NH_3_ and CO_2_, an important mechanism in soil to regulate N availability to plants [86, 87].

Catalase enzymes, on the other hand, decompose organic materials in the soil to a form that plants can use [88]. We found that intercropping had stronger urease and catalase activity than mono-cropping because of the high SOM content. Our findings are consistent with the findings of [13] and [78], who discovered significant changes in soil enzymes during intercropping systems. Changes in the soil enzyme activity have a direct impact on soil nutrient cycling, which in turn benefits the ecosystem, plant development and yield, and overall soil nutrients [89–92]. Thus, suggesting that intercropping maize with soybean can make significant changes to soil physical and chemical characteristics and available enzyme activities, which in turn improves the soil nutrients availability and plant nutrients uptake, thereby enhancing the crop yield.

## Conclusion

The results of this study indicates that maize-soybean intercropping at optimal nitrogen fertilization considerably enhanced the grain yield, biomass dry matter, and 1000-grain weight of maize crop as compared with mono-cropping. In addition, this maize-soybean intercropping system also boosted the plant nutrients content (i.e., N, P, K, Ca, Fe, and Zn). Moreover, maize/soybean intercropping also improved the soil-accessible nutrients and changed the soil enzymatic (i.e., PT, SC, AP, UE, and CT) activities, which adds to improved soil physio-chemical properties, and ultimately crop yields. This shows that maize-soybean intercropping with adequate nitrogen fertilization could boost plant nutrient content by modulating the soil’s physio-chemical characteristics and enzymatic activities, hence improving yield. Adopting intercropping as an agricultural method over mono-cropping may thus be a superior option for yield gains and plant-soil nutrient enhancement.

## Data Availability

No datasets were generated or analysed during the current study.
